# Tunneled catheter-related bacteremia in hemodialysis patients: incidence, risk factors and outcomes. A 14-year observational study

**DOI:** 10.1007/s40620-022-01408-8

**Published:** 2022-08-17

**Authors:** Marina Almenara-Tejederas, María A. Rodríguez-Pérez, María J. Moyano-Franco, Marina de Cueto-López, Jesús Rodríguez-Baño, Mercedes Salgueira-Lazo

**Affiliations:** 1 Nephrology Unit, Hospital Universitario Virgen Macarena, Seville, Spain; 2 Infectious Diseases and Microbiology Unit, Hospital Universitario Virgen Macarena, Seville, Spain; 3 Department of Medicine, University of Sevilla, Seville, Spain; 4 Biomedicine Institute of Seville (IBiS), CSIC, Centro de Investigación en Red en Enfermedades Infecciosas (CIBERINFEC), Seville, Spain; 5 Biomedicine Institute of Seville (IBiS), Biomedical Engineering Group, Center for Biomedical Research Network in Bioengineering Biomaterials and Nanomedicina (CIBER-BBN), Seville, Spain

**Keywords:** Bacteremia, Bloodstream, Hemodialysis, Tunneled catheter

## Abstract

**Background:**

Tunneled catheter-related bacteremia represents one of the major complications in patients on hemodialysis, and is associated with increased morbidity and mortality. This study aimed to evaluate the incidence of tunneled catheter-related bacteremia and, secondly, to identify possible factors involved in the first episode of bacteremia.

**Methods:**

This is a retrospective study of all tunneled catheters inserted between 1 January, 2005 and 31 December, 2019. Data on patients with a tunneled catheter were analyzed for comorbidities, catheter characteristics, microbiological culture results and variables related to the first episode of bacteremia. Patient outcomes were also assessed.

**Results:**

In the 14-year period under study, 406 tunneled catheters were implanted in 325 patients. A total of 85 cases of tunneled catheter-related bacteremia were diagnosed, resulting in an incidence of 0.40 per 1000 catheter days (81.1% after 6 months of implantation). The predominant microorganisms isolated were Gram-positive organisms: *Staphylococcus epidermidis* (48.4%); *Staphylococcus aureus* (28.0%). We found no significant differences in time to catheter removal for infections or non-infection-related reasons. The jugular vein, the Palindrome® catheter, and being the first vascular access were protective factors for the first episode of bacteremia. The 30-day mortality rate from the first tunneled catheter-related bacteremia was 8.7%.

**Conclusions:**

The incidence of bacteremia in our study was low and did not seem to have a relevant impact on catheter survival. *S. epidermidis* was the most frequently isolated microorganism, followed by *S. aureus*. We identified Palindrome® catheter, jugular vein, and being the first vascular access as significant protective factors against tunneled catheter-related bacteremia.

**Graphical abstract:**

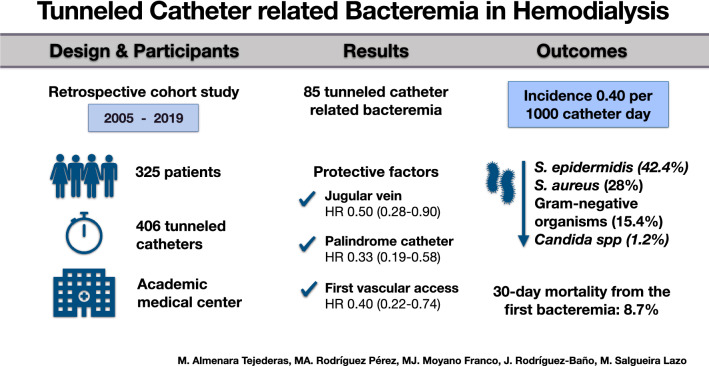

**Supplementary Information:**

The online version contains supplementary material available at 10.1007/s40620-022-01408-8.

## Introduction

Bloodstream infection, one of the major complications in patients on hemodialysis with prolonged central venous catheter dependence, is associated with increased risk of systemic infectious complications, hospitalizations, and death. Although guidelines recommend the use of arteriovenous accesses, the incidence of hemodialysis patients with tunneled catheter in Seville increased from 4.5% in 2015 to 44.6% in 2020 [1].

Available studies report an incidence ranging from 0.5 to 5.5 tunneled catheter-related bacteremia per 1000 days [2–6]. The infectious complications appear especially in the first 3–6 months of the life of the tunneled catheter [7]**.** Various approaches minimizing bacteremia and promoting infection prophylaxis are available, but the optimal combination of strategies is currently not well defined. Our study aimed to describe the protocol we use in our center and to evaluate the incidence of tunneled catheter-related bacteremia through a retrospective analysis of all tunneled catheters inserted over a period of 14 years. A secondary objective was to identify the possible factors involved in the bacteriemia of these hemodialysis patients.

## Methods

### Study design, inclusion/exclusion criteria and ethics

We performed a retrospective cohort study of adult patients in whom a tunneled catheter for hemodialysis was implanted between 2005 and 2019 at Hospital Universitario Virgen Macarena, a tertiary teaching center serving a population of 450,000 inhabitants in Seville, Spain. Patients transferred to, or with clinical follow-up at, another hospital area were excluded.

The tunneled catheters were inserted by nephrologists following a preimplantation protocol agreed upon with the Infectious Diseases Service, and is summarized herein (supplementary material, Table 1). Prior to catheter insertion, patients were screened for *Staphylococcus aureus* colonization and, if colonized, treated with intranasal mupirocin every 8 h for 5 days. Screening for *S. aureus* is also performed on all patients both at the start of hemodialysis and annually. All patients with a positive result are treated as explained in the text, repeating a second nasal exudate 7 days after finishing treatment. During the mupirocin treatment in colonized patients and in the hours prior to the tunneled catheter implantation, all patients showered using a 4% chlorhexidine soap solution.

The catheter insertion site was prepared using standard sterile procedures. After instillation of local anesthesia, the vein was accessed under direct sonographic guidance and the catheter was tunneled subcutaneously. Finally, the tunneled catheter was locked with citrate (Citra-Lock®) and used 24 h after implantation.

Patients were followed up from tunneled catheter insertion until the study end date (December 31, 2020), tunneled catheter-related bacteremia, or died. Whenever tunneled catheter-related bacteremia was suspected, two blood cultures were drawn, which consisted of a peripheral drawn blood sample and a second sample obtained from the tunneled catheter. In those patients in whom it was not possible to obtain peripheral vein blood cultures, catheter-related bacteremia was defined if the patient had compatible symptoms, with a positive blood culture from the catheter, and without evidence of another source of infection.

The study adhered to the guidelines laid out by the Declaration of Helsinki and the Declaration of Istanbul. The investigation was approved by the local Ethical Committee of Hospital Universitario Virgen Macarena. The need to obtain informed consent was waived because of the retrospective nature of the study.

### Variables and follow-up

The following demographic and clinical data were collected at the time of tunneled catheter implantation: age, sex, diabetes, hypertension, immunosuppression, cause of end-stage renal disease. Note that we describe the characteristics of the patients, even though they may have had more than one episode of tunneled catheter-related bacteremia. Regarding the characteristics of the catheter, our data refer to the first catheter implanted or the catheter with infection in those patients with bacteremia. The catheter-related parameters we considered were; medical reason for implantation, site of catheter insertion, brand name. Variables related to the first episode of bacteremia were also recorded.

### Tunneled catheter-related bacteremia definition

The primary outcome of interest was tunneled catheter-related bacteremia occurrence. Tunneled catheter-related bacteremia was defined as the presence of fever or systemic signs of infection in a patient with a tunneled catheter, with no other evident infectious source, with detection of identical microorganisms in blood cultures obtained from the peripheral vein and the catheter. The differential time to positivity was used to confirm diagnosis of catheter-related bacteremia. A differential time to positivity of ≥ 120 min of blood cultures obtained from the catheter and a peripheral vein was considered diagnosis for catheter-related bacteremia [8]. Early bacteremia was defined as that detected in the first 90 days after tunneled catheter implantation. Secondary outcomes included time to first tunneled catheter-related bacteremia, etiological agents, and potential influencing factors.

### Statistical analysis

Tunneled catheter-related bacteremia incidence density was calculated as the number of new episodes per 1000 catheter-days. Continuous variables were expressed as mean and 95% confidence interval (CI) or median and the first and third quartile (Q1–Q3)**,** as appropriate. They were compared using Student’s *t* test or the Wilcoxon test according to their distribution. Normal distribution of the data was examined by the Shapiro–Wilk-test. Categorical variables were summarized as counts and percentages and were evaluated using Chi-squared or Fisher’s exact test.

We used the multivariable Cox regression adjusting for baseline confounders to study the impact of our variables on risk of the first bacteremia of each included patient. All variables with a *p* value of < 0.2 on the univariate analysis were included for evaluation in the multivariate analysis. For survival analyses, we generated Kaplan–Meier curves; comparison was done using the log-rank test. Significance level was set at 0.05. Statistical analyses were performed using SPSS version 26.

## Results

### Patients’ features and tunneled catheter-related bacteremia incidence density

A total of 462 tunneled catheters were implanted in 381 patients over a period of 14 years. Fifty-six patients were excluded due to their follow-up in another hospital area. To our knowledge, none of them had tunneled catheter-related bacteremia. Therefore, we included 325 patients with a total of 406 tunneled catheters (Supplementary Material, Fig. 1).

For our study, we analyzed the individual characteristics of the patients. The median age of the patients was 67 (55–74) years; 179 (55.1%) were men, 154 (47.4%) had diabetes mellitus, and 292 (89.9%) had hypertension. Diabetic kidney disease was the major cause of chronic kidney disease (CKD). In 159 (48.9%) cases, the patients were on immunosuppressants; specifically, 32 (9.9%) had received a renal transplantation. Patients’ characteristics are summarized in Table 1.

**Table 1:** Clinical and demographic characteristics of the 325 patients with tunneled catheters included in the study

Characteristics	Median (Q1–Q3)
Age (years)	67 (55–74)
Hemodialysis time (days)	53
Characteristics	*N* (%)
Male sex	179 (55.1)
Diabetes mellitus	154 (47.4)
Hypertension	292 (89.8)
Immunosuppression	159 (48.9)
HIV infection	1 (0.3)
Malignancy	66 (20.3)
Corticosteroids	35 (10.8)
Inmunosupressant drugs	42 (13.0)
Kidney transplant	32 (9.9)
Hematologic disease	33 (10.2)
Monoclonal gammopathy	13 (39.4)
Leukemia	5 (15.2)
Lymphoma	4 (12.1)
Myeloma	8 (24.2)
Myeloproliferative syndrome	3 (9.1)
COPD/asthma	33 (10.2)
Chronic liver disease	28 (8.6)
Kidney transplant	32 (9.9)
Chronic kidney disease etiology	
Diabetic	70 (21.5)
Vascular	48 (14.8)
Glomerulopathy	55 (16.9)
Tubulointerstitial nephropathy	53 (16.3)
Vasculitis	8 (2.5)
Polycystic kidney disease	10 (3.1)
Others	26 (8.0)
Unrelated etiology	55 (16.9)
Implantation vein	
Jugular	275 (84.6)
Subclavian	47 (14.5)
Femoral	3 (0.9)
Implantation side	
Right	294 (90.5)
TC brandmark	
Palindrome®	192 (59.1)
Implantation cause	
Vascular access depletion	85 (27.0)
Prior access dysfunction	90 (28.6)
First vascular access	100 (31.7)
Arteriovenous fistula contraindication	40 (12.7)
Removal cause	
Infection (tunnelitis/TCRB)	26 (17.9)
TC dysfunction	24 (16.6)
Useful arteriovenous fistula	48 (33.1)
Hemodialysis exit	47 (32.4)

*COPD* chronic obstructive pulmonary disease, *TC* tunneled catheter, *TCRB* tunneled catheter-related bacteremia

Regarding the characteristics of the catheters, we referred to the first catheter implanted or the catheter with infection in those patients with tunneled catheter-related bacteremia. The causes of tunneled catheter implantation were vascular access depletion (exhausted all options for creation of a permanent vascular access) (85, 27.0%), previous vascular access dysfunction (90, 28.6%), or the need for hemodialysis without a vascular access available (140, 44.4%). The internal jugular vein was the site of placement in 275 (84.6%) patients; the right side was chosen in 294 (90.5%) patients.

Of 325 patients, 57 (17.5%) suffered at least one episode of tunneled catheter-related bacteremia. Among these, 40 (70.1%) had one episode, 11 (19.3%) had 2, 4 (7.0%) had 3, 1 (1.8%) had 5 and 1 (1.8%) had 6. Therefore, 85 cases of tunneled catheter-related bacteremia were diagnosed, resulting in an incidence of 0.40 per 1000 catheter days. Table 2 shows the annual tunneled catheter-related bacteremia rate and the incidence in absolute numbers. The median time from tunneled catheter implantation to first bacteremia was 452 days (155–706).

**Table 2:** Yearly tunneled catheter-related bacteremia incidence and rates

	Number of implanted TCs	Number of functioning TCs	TC days	Number of TCRB	TCRB rate	Cummulative TCRB rate	Number of TCs removed
2005	8	8	859	0	0	0	0
2006	19	27	5651	2	0.35	0.31	2
2007	21	46	11,205	5	0.44	0.39	15
2008	27	58	11,717	9	0.76	0.54	19
2009	16	47	11,204	3	0.26	0.47	16
2010	8	39	11,397	9	0.79	0.54	11
2011	12	40	8239	12	1.46	0.66	18
2012	13	35	9589	6	0.62	0.66	4
2013	25	56	13,009	5	0.38	0.61	17
2014	28	67	15,091	6	0.40	0.58	24
2015	27	70	16,823	4	0.24	0.53	17
2016	39	92	22,061	7	0.32	0.50	27
2017	44	109	24,112	10	0.41	0.48	44
2018	48	112	24,159	3	0.12	0.44	37
2019	71	146	29,097	4	0.14	0.40	66
Total	**406**	**–**	**214,213**	**85**	**–**	**0.40**	**317**

The last row of bold referring to the total numbers during the study

*TC* tunneled catheter, *TCRB* tunneled catheter-related bacteremia

### Etiology of tunneled catheter-related bacteremia

The majority (83.4%) of tunneled catheter-related cases of bacteremia were caused by gram-positive organisms, including *Staphylococcus epidermidis* (48.4%) and *S. aureus* (28.0%). Methicillin-resistant strains accounted for 12.5% of *S. aureus* isolates (Table 3). A variety of gram-negative bacteria accounted for 15.5% of episodes. Bacteremia secondary to *Candida *spp. only affected 1.2% of patients. These percentages were similar when only the first episode in each patient was considered: *S. epidermidis* accounted for 42.1%, followed by *S. aureus* (31.6%) (Table 3).

**Table 3:** Etiology of tunneled catheter-related bacteremia

Microorganisms, *n* (%)	All TCRB	1st TCRB	2nd TCRB	3rd TCRB	Other TCRB
*S. aureus,* methicillin-susceptible	21 (24.5)	15 (26.3)	5 (29.4)	1 (16.7)	–
*S. aureus,* methicillin-resistant	3 (3.5)	3 (5.3)	–	–	–
*S. epidermidis*	36 (42.4)	24 (42.1)	6 (35.3)	2 (33.3)	3 (80.0)
*S. warneri*	2 (2.4)	1 (1.8)	1 (5.9)	–	–
*S. hominis*	1 (1.2)	1 (1.8)	–	–	–
*Streptococcus viridans*	1 (1.2)	1 (1.8)	–	–	–
*Streptococcus bovis*	1 (1.2)	1 (1.8)	–	–	–
*Corynebacterium striatum*	3 (3.5)	2 (3.5)	–	–	–
*Enterococcus faecalis*	3 (3.5)	–	1 (5.9)	2 (33.3)	1 (20.0)
*Pantoea agglomerans*	2 (2.4)	2 (3.5)	–	–	–
*Serratia marcensens*	4 (4.6)	3 (5.3)	–	1 (16.7)	–
*Serratia rubidae*	1 (1.2)	1 (1.8)	–	–	–
*Pseudomona aeruginosa*	2 (2.4)	2 (3.5)	–	–	–
*Klebsiella pneumoniae*	1 (1.2)	1 (1.8)	–	–	–
*Enterobacter cloacae*	1 (1.2)	–	1 (5.9)	–	–
*Proteus mirabilis*	2 (2.4)	–	2 (11.8)	–	–
*Candida spp*	1 (1.2)	–	1 (5.9)	–	–

*TCRB* tunneled catheter-related bacteremia, *MSSA* *methicillin*-susceptible *Staphylococcus aureus,* *MRSA* *methicillin-resistant Staphylococcus aureus*, *spp* several species

Only 12 (14.2%) tunneled catheter-related episodes of bacteremia occurred in the first 90 days after catheter implantation. There were 4 episodes of bacteremia (4.7%) between 90 days and 6 months after tunneled catheter implantation. The remaining 69 (81.1%) episodes occurred more than 6 months after catheter implantation.

### Risk factors for first tunneled catheter-related bacteremia

The univariate association of different variables with the risk of first tunneled catheter-related bacteremia is shown in Table 4. Patients with bacteremia more frequently had a non-Palindrome® catheter and vascular access depletion. The median time to first bacteremia differed depending on vein, catheter type and indication for implantation (Supplementary Material, Fig. 2).

**Table 4:** Clinical and demographic characteristics of the patients included in the study according to the diagnosis or not of tunneled catheter-related bacteremia

Characteristic	No TCRB (*n* = 268)	TCRB (*n* = 57)	*p* value
	Median (Q1–Q3)	Median (Q1–Q3)	
Age (years)	67 (55–75)	66 (54–74)	0.43
Hemodialysis time (days)	43 (8–287)	181 (51–932)	0.24
	*N* (%)	*N* (%)	
Male sex	149 (55.6)	30 (52.6)	0.79
Diabetes mellitus	133 (49.6)	21 (36.8)	0.1
Hypertension	239 (89.2)	53 (93.0)	0.53
Immunosuppression	130 (48.5)	29 (50.9)	0.85
HIV infection	1 (0.4)	0	0.82
Malignancy	52 (19.4)	14 (24.6)	0.48
Corticosteroids	33 (12.3)	2 (3,5)	0.08
Immunosuppressive drugs	35 (13.1)	7 (12.3)	1.00
Hematologic disease	30 (11.2)	3 (5.3)	0.26
COPD/asthma	30 (11.2)	3 (5.3)	0.26
Chronic liver disease	25 (9.3)	3 (5.3)	0.46
Kidney transplant	23 (8.6)	9 (15.8)	0.16
CKD etiology			
Diabetic	56 (20.9)	14 (24.6)	0.15
Vascular	39 (14.6)	9 (15.8)	
Glomerulopathy	46 (17.2)	9 (15.8)	
Tubulointerstitial neph	38 (14.2)	15 (26.3)	
Vasculitis	7 (2.6)	1 (1.8)	
ADPKD	10 (3.7)	0	
Others	25 (9.3)	1 (1.8)	
Not affiliated	47 (17.5)	8 (14.0)	
Implantation vein—jugular	233 (86.9)	38 (66.7)	< 0.001
Implantation side—right	243 (90.7)	48 (84.2)	0.95
TC brandmark Palindrome®	173 (64.6)	24 (42.1)	< 0.001
Vascular access depletion	60 (23.1)	25 (45.5)	< 0.001

*COPD* chronic obstructive pulmonary disease, *TC* tunneled catheter, *TCRB* tunneled catheter-related bacteremia

The jugular vein in comparison with the femoral and subclavian veins, the use of the Palindrome® catheter, and being the first vascular access (versus depletion of vascular access) were protective factors for the presentation of the first episode of tunneled catheter-related bacteremia. The hazard ratio was 0.50 (0.28–0.90) for the jugular vein, 0.33 (0.19–0.58) for Palindrome® catheter and 0.40 (0.22–0.74) for the first vascular access (*p* = 0.04). We found no significant influence of other studied parameters on the risk of bacteremia. Multivariate analysis showed that the use of the Palindrome® catheter was a protective factor (HR 0.17; 95% CI 0.10–0.31; *p* < 0.001), as was the use of a tunneled catheter as the first vascular access (HR 0.41; 0.23–0.75; *p* = 0.004) (Table 5).

**Table 5:** Variables included in the multivariable analysis

Variables	No TCRB (*n* = 268)	TCRB (*n* = 57)		Multivariate analysis	
	Median (Q1–Q3)	Median (Q1–Q3)	*p* value	Hazard ratio	*p* value
TC brandmark Palindrome®	173 (64.6)	24 (42.1)	< 0.001	0.17 (0.10–0.31)	< 0.001
First vascular access	60 (23.1)	25 (45.5)	< 0.001	0.41 (0.23–0.75)	0.004

*TC* tunneled catheter, *TCRB* tunneled catheter-related bacteremia

### Management of tunneled catheter and outcomes

A total of 145 patients had the tunneled catheter removed or exchanged during the study due to different causes. The main reasons for catheter removal were the adequate development and use of the arteriovenous fistula (48, 33.1%) and discharge from hemodialysis due to recovery of renal function, transfer to peritoneal dialysis or renal transplantation (47, 32.4%) (Table 1). Only 26 (17.9%) patients underwent tunneled catheter removal due to bacteremia at a median of 4.8 (1.0–8.0) days from the episode. This means that the tunneled catheter was removed in 30.5% of the 85 bacteremias detected during the study.

The median time to catheter removal for non-infection-related reasons was 448 (185–910) days, without significant differences vs the tunneled catheter-related bacteremia group [430 (116–695)] (*p* = 0.83).

During the study period, a total of 168 (51.7%) patients died of different causes. Among the 57 patients with tunneled catheter-related bacteremia, five died within 30 days after the first episode of bacteremia, thus resulting in a 30-day mortality rate from the first episode of tunneled catheter-related bacteremia of 8.7%. The microorganisms that were responsible for the tunneled catheter-related bacteremia were *S. epidermidis* (2), *S. aureus* (2) and *Corynebacterium *spp (1). If we consider any episode of bacteremia, two further patients died within 30 days. The microorganisms involved were *S. epidermidis* (1) and *S. aureus* (1). In our study, a total of 7 (12.2%) patients died from bacteremia. Deaths occurred between 2010 and 2015. The characteristics of the patients who died during the study are summarized in Supplementary Material, Table 2.

## Discussion

Tunneled catheter-related bacteremia is a serious complication in patients undergoing hemodialysis, and is associated with increased risk of morbidity and mortality [9]. Our study investigated the incidence, causative microorganisms and factors associated with catheter-related bacteremia in hemodialysis patients using a tunneled catheter. Overall, 1 out of every 5–6 patients experienced at least one tunneled catheter-related episode of bacteremia, with a rate of 0.40 per 1000 catheter days. *S. epidermidis* caused the majority of episodes. Most cases of tunneled catheter-related bacteremia occurred more than 6 months after catheter implantation. We found that placement in the jugular vein, use of the Palindrome® catheter, and being the first vascular access were protective factors for tunneled catheter-related bacteremia.

The use of tunneled catheters continues to increase in most nephrology units. In the province of Seville, according to data from the SICATA registry [1], the incidence of hemodialysis patients with tunneled catheter increased from 4.5% in 2015 to 44.6% in 2020 [1] (Supplementary Material, Table 3). It should be noted that our study reports data from a single hospital (Table 2), which covers approximately one third of the population of the province. However, the remarkable increase in the use of tunneled catheters in recent years in Seville was observed in both registries and could be mainly due to two reasons. On the one hand, the great difficulty in achieving a functioning internal vascular access at the start of hemodialysis due not only to the current profile of the hemodialysis patient, but also to the lack of vascular surgical activity in some centers. On the other hand, better patient care in the pre-dialysis phase and better training of nephrologists in the implantation of this type of catheter has made it possible to reduce the use of temporary catheters in incident hemodialysis patients.

The incidence of tunneled catheter-related bacteremia in our study was relatively lower than what has been reported in the literature (0.5–5.5 events/1000 catheter-days) [2–6]. Comparing our data with the classification of Beathard and Urbanes, our protocol achieved an incidence of tunneled catheter-related bacteremia corresponding to the excellent level [4]. There is considerable variation in infection prevention and control practices, so the optimal bundle of prophylactic measures remains undefined. However, the use of aseptic measures in a standardized protocol has been shown to reduce the rate of catheter-related bacteremia [4, 10, 11]. This is consistent with our clinical practice and leads to some observations we believe to be significant.

The Spanish Clinical Guidelines on Vascular Access for Haemodialysis [12] do not recommend the screening and decolonization of *S. aureus* nasal carriage due to the development of resistance. However, several studies showed that *S. aureus* colonization increases the risk of infection [13, 14], associating a worse prognosis in cases of methicillin-resistant *S. aureus* (MRSA) [15]. A recent study by Vanegas et al. estimated that the risk of bacteremia caused by *S. aureus* was 5.90 times higher in colonized patients [5]. On the other hand, intranasal mupirocin prophylaxis has been associated with a significant reduction in the incidence of *S. aureus* bacteremias [16]. This prophylactic procedure is included in our protocol and could be related to the low reported rate of tunneled catheter-related bacteremia. Although mupirocin treatment must be administered with caution due to the development of resistance, the use of *S. aureus* decolonization protocols in patients with tunneled catheter should be revised.

The chlorhexidine body wash is also included as a prevention strategy in our protocol. This measure has not been studied in hemodialysis patients. However, in 2 meta analyses published in 2016, a reduction in catheter-related bacteremia was observed by bathing with chlorhexidine in intensive care units [17, 18]. Antibiotic prophylaxis before insertion of the central venous catheter has been analyzed in cancer patients and in those requiring enteral nutrition, with discordant results [19–21]. There are no data from recent studies in hemodialysis patients. Although anticoagulant or preventive antibiotic locks have been used in several studies, resulting in a reduction of infection [16], there is also no updated evidence to support them [12]. Their use should be limited to necessary cases because of the emergence of antimicrobial resistance.

With regard to the microorganisms involved in tunneled catheter-related bacteremia, our study findings are relatively consistent with the literature [3, 22]. It has been estimated that coagulase-negative staphylococci and *S. aureus* account for 60–80% of cases in most studies [9, 22, 23]. However, many of these reports identified *S. aureus* as the most frequent microorganism, while *S. epidermidis* was predominant in our cohort, causing 48.4% of cases. Our rate of MRSA was lower than previously reported [2, 3, 24]. The lower incidence of *S. aureus* infection, with a low percentage of MRSA, may be related to the strict aseptic measures and *S. aureus* screening and decolonization used in our protocol.

The risk of tunneled catheter-related bacteremia increased with the duration of catheter dependence [5]. Our findings are consistent with the literature: the risk of tunneled catheter-related bacteremia was particularly high after 6 months of the life of the catheter, while it was rare in the first three months. We must perhaps improve our long-term preventive strategies.

Regarding the protective factors found in our study, the association of catheter placement in the jugular vein with lower risk of infection is consistent with previous studies in which the femoral vein was found to be a risk factor for bacteremia as compared to subclavian and internal jugular sites [2, 5].

With regard to the Palindrome® catheter, this is a catheter with a symmetrical spiral end tip and laser-cut side slots that has the potential, theoretical benefit of improved rheologic performance, and reduced propensity for both thrombosis and tunneled catheter-related bacteremia [25, 26]. However, the comparative studies between different types of catheters have not demonstrated significant differences in the risk of infection [12, 27–29]. A comparative analysis of the different types of tunneled catheters was not included among the initial objectives of our study. In addition, there was a higher percentage of jugular vein catheters and first vascular accesses among patients with a Palindrome® catheter. For these reasons, the protective factor of the Palindrome® catheter reported in our study should be viewed with caution.

Finally, the implantation of the tunneled catheter as the first vascular access (versus the indication of implantation due to depletion of vascular access) was found to be a protective factor. This could be because these patients with vascular access depletion have greater vascular complications and overall comorbidity.

It is evident that the risk of tunneled catheter-related bacteremia increases with time [23, 30]. In our study, the Kaplan–Meier curve showed a linear rate in the occurrence of bacteremia during the first 1000 days of follow-up, which then flattened. We used Cox regression models in order to control the effect of time at risk in the estimation of the influence of other variables. A higher incidence of tunneled catheter-related bacteremia was also observed in 2011. However, we could not see an outbreak caused by a specific pathogen; no patients who experienced tunneled catheter-related bacteremia in 2011 had a Palindrome® catheter, and the percentage of vascular access depletion as a cause of implantation was significantly higher than those with bacteremia in other years. These factors, together with a possible, reduced adherence to the rules for the management of infection might explain the higher number of tunneled catheter-related episodes of bacteremia that year.

Our analysis revealed that 39.5% of the episodes of tunneled catheter-related bacteremia required catheter removal due to the infection; this accounted for 17.9% of the total catheters removed during the study, and it is slightly lower than that described in previous studies. In the study of Marr et al. [3], 41 patients (40%) developed 62 episodes of bacteremia. Twenty-four catheters (39%) were removed immediately, and 38 (61%) were left in place during treatment. Only 12 (32%) of the 38 catheters were salvaged successfully. Of the 62 episodes of tunneled catheter bacteremia, 50 (80.6%) catheters were removed due infection (compared with 30.5% in our study). Shingarev et al. [30] did not report the total number of bacteremia episodes in their study. However, they reported that 206 of the 472 tunneled catheters were removed non-electively (55% due to dysfunction and 45% due to catheter-related bacteremia).

During the study period, 8.7% of patients died within 30 days after the first episode of tunneled catheter-related bacteremia. Mortality following bacteremia has received relatively little attention in the available literature. Spanish and European guidelines have reported higher risk of bacteremia in patients with catheters, with increased morbidity and mortality compared to the use of fistulas, but they did not collect information on mortality rate [12, 31]. There are no clinical trials reporting mortality rates secondary to tunneled catheter-related bacteremia; the few observational studies published highly variable mortality rates. Nelveg-Kristensen et al. [32] reported a death rate after bacteremia of 13.8% for a cohort of 2646 incident patients on renal replacement therapy. If we focus on hemodialysis catheter-dependent patients, Vanegas et al. described that 8% (*n* = 4) of the patients with bacteremia died because of the infection during the hospital stay [5]. Two more recent studies focused on mortality in patients with tunneled catheter-related bacteremia. In the 3-year study of Shahar et al. [2], patients with catheter-related bloodstream infections (or catheter colonization) were included, and two out of 175 patients died (1.1%). Farrington et al. presented a series of 289 patients with tunneled catheter-related bacteremia, of whom 1% died directly because of bacteremia or its metastatic complications [9]. Although mortality in our analysis is considerable, and higher than that reported in these two studies, the results cannot be directly compared for different reasons. Shahar et al. [2] included patients with colonization of the catheter who normally present asymptomatic or with mild symptoms. Farrington et al. [9] excluded recurrent catheter-related bacteremia (defined as a second infection with the same organism occurring less than 3 months earlier) in their analysis. In addition, the sample size and the study period were greater in our series. We must also emphasize that all episodes of bacteremia in our study occurred before 2015, thus if we analyzed mortality in the last 5–7 years, it would be zero. The improvement in hospital care, the promotion of diagnosis and early initiation of antibiotic therapy, and the greater training of our professionals have possibly led to the progressive decrease in mortality in patients with tunneled catheter-related bacteremia.

Our study has limitations that should be considered when interpreting the results. It was a retrospective, single hospital study, which limits its generalizability to other settings. In addition, we did not directly compare patients with a tunneled catheter to those with other forms of vascular access. Some strengths include a careful follow-up of the patients, a large population and data from a specific preventive protocol.

In conclusion, the incidence of tunneled catheter-related bacteremia in our study was low and showed a clear increase after 6 months of tunneled catheter implantation. Compliance with a rigorous catheter implantation and management protocol could reduce the risk of early bacteremia. *S. epidermidis* was the most frequently isolated microorganism in our region, followed by *S. aureus*. The median time to tunneled catheter removal for non-infection-related reasons did not differ from the bacteremia group, indicating a non-relevant impact of bacteremia on catheter survival. We identified Palindrome® catheter, jugular vein, and being the first vascular access as significant protective factors for tunneled catheter-related bacteremia. Further randomized studies are needed to directly compare the different types of catheters and determine other possible risk factors for tunneled catheter-related bacteremia.

## Supplementary Information

Below is the link to the electronic supplementary material.

Supplementary file1 (DOCX 917 kb)
