# Supplementary material for: Tunneled catheter-related bacteremia in hemodialysis patients: incidence, risk factors and outcomes. A 14-year observational study
Source: J Nephrol. 2022 Aug 17;36(1):203–12. doi: 10.1007/s40620-022-01408-8 (PMC9895018; doi:10.1007/s40620-022-01408-8)
Supplement: Supplementary file 1 — Supplementary file1 (DOCX 917 kb) [file 40620_2022_1408_MOESM1_ESM.docx]

Table 1. Tunneled catheter preimplantation and management protocol.

| **Prior to admission for catheter implantation** | **Screening for *Staphylococcus aureus* colonization by nasal exudate.**  **Colonized patients (positive result):**   - Treatment with intranasal mupirocin every 8 hours for 5 days - Shower using a 4% chlorhexidine soap solution for 5 days - Second nasal exudate 7 days after finishing treatment. if it remains positive, the patient is referred to the Infectious Diseases unit for evaluation of oral treatment. |
| --- | --- |
| **Perioperative period** | **Shower using a 4% chlorhexidine soap solution**  **Administration of Cefazolin (20 mg/Kg, maximum 2g) just before the procedure in patients at risk of bacteremia (carriers of temporary or permanent catheter).**   - Administration of vancomycin in carriers of MRSA or allergic to beta-lactams   **Preparation of the insertion area with sterile measures**  **Tunneled catheter implantation by nephrologists**   - After instillation of local anesthesia, the vein was accessed under direct sonographic guidance and the catheter was tunneled subcutaneously.   **Tunneled catheter lock with citrate (Citra-Lock®)** |
| **First hours after implantation of the tunneled catheter** | **First use 24 hours after implantation** |
| **Tunneled catheter care in hemodialysis sessions** | **Disinfection of the catheter with 2% aqueous Chlorhexidine** before each hemodialysis session and each time it is manipulated  **Removal of the fixation points of the tunneled catheter to**  **10-14 days from implantation.**  **Cures of the catheter orifice every 5-7 days from the removal of the fixation points.**   - Surveillance of insertion should be carried out at each session, changing the dressing whenever it is moist, stained, or peeling, or that the patient has tenderness, fever, or some other sign of infection |

Table 2. Clinical and demographic characteristics of the patients who died during the study.

| **Characteristics** | **Median (Q1-Q3)** |
| --- | --- |
| Age (years) | 71 (55-74) |
| Hemodialysis time (days) | 1071 (132-1369) |
| **Characteristics** | **N (%)** |
| Male sex | 4 (57.1) |
| Diabetes mellitus | 2 (28.6) |
| Hypertension | 6 (85.7) |
| Immunosuppression  Inmunosupressants drugs  Hematologic disease | 4 (57.1)  3 (42.9)  1 (14.3) |
| COPD/asthma | 0 |
| Chronic liver disease | 0 |
| Kidney transplant | 1 (14.3) |
| Chronic kidney disease etiology  Diabetic  Glomerulopathy  Tubulointerstitial nephropathy  Vasculitis  Unrelated etiology | 1 (14.3)  2 (28.6)  2 (28.6)  1 (14.3)  1 (14.3) |
| Implantation vein  Jugular  Subclavian  Femoral | 2 (28.6)  4 (57.1)  1 (14.3) |
| Implantation side  Right | 6 (85.7) |
| TC brandmark  Palindrome® | 2 (28.6) |
| Implantation cause  Vascular Access depletion  Prior access dysfunction  First vascular access  Arteriovenous fistula contraindication | 2 (33.3)  2 (33.3)  1 (14.3)  2 (33.3) |

COPD: chronic obstructive pulmonary disease; TC: tunneled catheter.

Table 3. Initial vascular access in incident hemodialysis patients in Seville

|  | Temporary catheter | Tunneled catheter | Arteriovenous fistula | Arteriovenous graft | Total |
| --- | --- | --- | --- | --- | --- |
| 2015 | 9 (48.8%) | 9 (4.5%) | 92 (44.7%) | 2 (0.6%) | 112 |
| 2020 | 68 (30.4%) | 100 (44.6%) | 55 (24.6%) | 1 (0.4%) | 224 |

Figure 1. Flow chart showing the selection process of the study population.

| 381 patients admitting from January 2005 to December 2019 |
| --- |

| Excluded (n=56)  Clinical follow-up in another health area |
| --- |

| 325 patients were included in analyses |
| --- |

406 tunneled hemodialysis catheters

Figure 2. Kaplan-Meier curves: probability of being free of bacteremia.


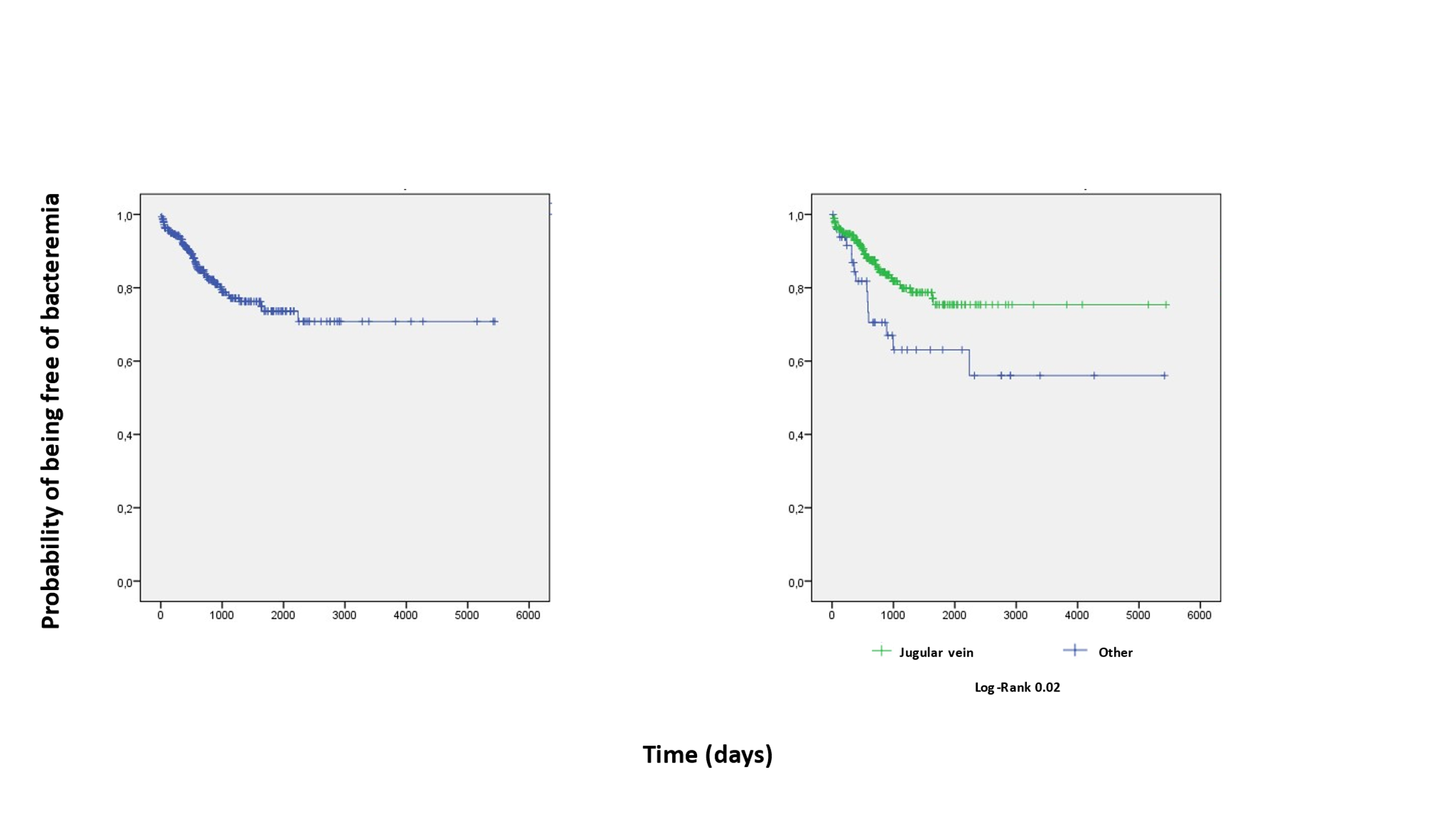


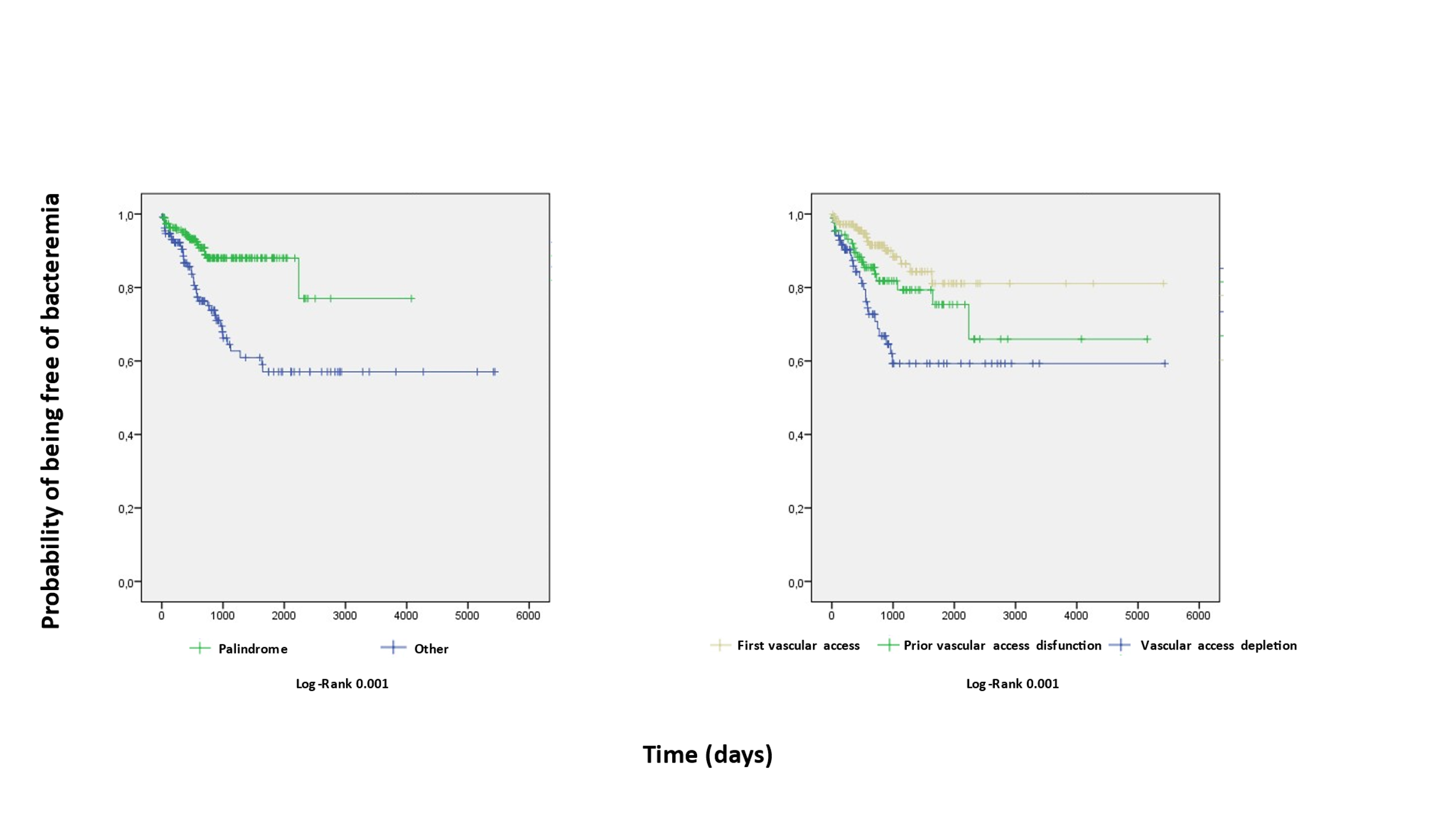


Legends of figure 1. Kaplan-Meier curves. Probability of being free of bacteremia was censored at time of death.

A. Kaplan-Meier curve showing time to first tunneled catheter related bacteremia in 325 included patients.

B. Kaplan-Meier curves showing tunneled catheter related bacteremia incidence in the jugular vein and other veins groups.

C. Kaplan-Meier curve of tunneled catheter related bacteremia-free survival for the Palindrome® and other catheters.

D. Kaplan-Meier curves by implantation cause: first vascular access, prior vascular access disfunction, and vascular access depletion.
